# Human Embryonic Stem Cell-Derived Wilson’s Disease Model for Screening Drug Efficacy

**DOI:** 10.3390/cells9040872

**Published:** 2020-04-02

**Authors:** Dongkyu Kim, Su-Bin Kim, Jung Lim Ryu, Heesu Hong, Jin-Hyuk Chang, Tack-Jin Yoo, Xiong Jin, Han-Jin Park, Choongseong Han, Beom Hee Lee, Jin-Ho Choi, Han-Wook Yoo, Jong-Hoon Kim, Dong-Hun Woo

**Affiliations:** 1 Department of Stem Cell Research, NEXEL Co., Ltd., 8th floor, 55, Magokdong-ro, Gangseo-gu, Seoul 07802, Korea; dkkim@nexel.co.kr (D.K.); subin0208@nexel.co.kr (S.-B.K.); fbwjdfla@naver.com (J.L.R.); inchoiminam@naver.com (H.H.); jhchang@nexel.co.kr (J.-H.C.); nexelceo@nexel.co.kr (C.H.); 2 Department of New Drug Development, NEXEL Co., Ltd., 8th floor, 55, Magokdong-ro, Gangseo-gu, Seoul 07802, Korea; yootackjin@nexel.co.kr (T.-J.Y.); xjin@nexel.co.kr (X.J.); 3 Predictive Model Research Center, Korea Institute of Toxicology, Daejeon 34114, Korea; hjpark@kitox.re.kr; 4 Department of Pediatrics, Asan Medical Center Children’s Hospital, University of Ulsan College of Medicine, Seoul 05505, Korea; bhlee@amc.seoul.kr (B.H.L.); jhc@amc.seoul.kr (J.-H.C.); hwyoo@amc.seoul.kr (H.-W.Y.); 5 Laboratory of Stem Cells and Tissue Regeneration, Department of Biotechnology, College of Life Sciences and Biotechnology, Science Campus, Korea University, 145 Anam-ro, Seongbuk-gu, Seoul 02841, Korea; jhkim@nexel.co.kr

**Keywords:** human embryonic stem cell, Wilson’s disease, CRISPR/Cas9, hepatocyte differentiation, copper toxicity, drug screening system

## Abstract

Human pluripotent stem cells (hPSCs) including human embryonic stem cells (hESCs) and human-induced pluripotent stem cells (hiPSCs) have been extensively studied as an alternative cellular model for recapitulating phenotypic and pathophysiologic characters of human diseases. Particularly, hiPSCs generated from the genetic disease somatic cells could provide a good cellular model to screen potential drugs for treating human genetic disorders. However, the patient-derived cellular model has a limitation when the patient samples bearing genetic mutations are difficult to obtain due to their rarity. Thus, in this study, we explored the potential use of hPSC-derived Wilson’s disease model generated without a patient sample to provide an alternative approach for modeling human genetic disease by applying gene editing technology. Wilson’s disease hPSCs were generated by introducing a R778L mutation in the *ATP7B* gene (c.2333G>T) using Clustered Regularly Interspaced Short Palindromic Repeats (CRISPR)/Cas9 system into wildtype hESCs. Established Wilson’s disease hESCs were further differentiated into hepatocyte-like cells (HLCs) and analyzed for disease phenotypes and responses against therapeutic agent treatment. R778L mutation in the *ATP7B* gene was successfully introduced into wildtype hESCs, and the introduction of the mutation neither altered the self-renewal ability of hESCs nor the differentiation capability into HLCs. However, R778L mutation-introduced HLCs exhibited higher vulnerability against excessive copper supplementation than wildtype HLCs. Finally, the applicability of the R778L mutation introduced HLCs in drug screening was further demonstrated using therapeutic agents against the Wilson’s diseases. Therefore, the established model in this study could effectively mimic the Wilson’s disease without patient’s somatic cells and could provide a reliable alternative model for studying and drug screening of Wilson’s disease.

## 1. Introduction

Wilson’s disease (Online Mendelian Inheritance in Man, OMIM #277900) is a genetic disorder in which excessive copper accumulates due to abnormal metabolism of copper [1,2]. Although copper is an essential trace element required for a healthy life, excessive copper should be excreted from the human body to maintain normal homeostasis. In Wilson’s disease patients, copper is not removed properly due to mutations in the *ATP7B* gene that encodes the copper transporting P-type ATPase, which results in damages in several organs [3,4].

The most affected organ in Wilson’s disease patient is the liver because it is the primary organ that encounters copper metabolism [5,6]. When the disease is diagnosed in the early phase, the important strategy for curing it is lowering the amount of copper level in the body in order to prevent the accumulation of excessive copper. Therefore, low copper diet and pharmacologic treatment with therapeutic agents are frequently utilized for lifelong treatment [7,8,9]. In severe cases, liver transplantation is considered as the last option [10,11]. Although current understanding on the pathophysiology of Wilson’s disease is well documented, a lot of study should be done to elucidate the curing process, selecting proper medicines and treatment methods. To achieve this, a reliable disease model that recapitulates Wilson’s disease is strongly required.

Human pluripotent stem cells (hPSCs) including human embryonic stem cells (hESCs) and human-induced pluripotent stem cells (hiPSCs) could provide an invaluable cell source to model human disease because their self-renewal ability and differentiation capability [12,13,14,15]. In fact, several studies demonstrated the hepatic differentiation and disease phenotypes of differentiated hepatocyte-like cells (HLCs) from the patient-derived hiPSCs for modeling Wilson’s disease [16,17,18]. However, there is a limitation in the modeling genetic disease including Wilson’s disease with patient somatic cell-derived hiPSCs due to the disease’s rarity.

Recently, a new gene-editing technology, Clustered Regularly Interspaced Short Palindromic Repeats (CRISPR)/Cas9 system, has been developed [19,20]. This technology enables an efficient and reliable method for precise genome editing (e.g., inserting and deleting specific DNA fragments, correction, or substitution of sequences) in mammalian cells [21]. In addition to this advantage, CRISPR/Cas9 system facilitates the modeling of human-inherited disorders in hPSCs by introducing specific site mutations in their genome [22,23,24].

In this study, we suggested a promising approach for modeling Wilson’s disease without patient samples by introduction of disease mutation in wildtype hESCs using gene-editing technology and demonstrated the effectiveness of the mutation introduced model by comparing with the same mutation bearing Wilson’s patient-derived model. This mutation-induced hESCs recapitulated the defects in copper-related phenotypes of Wilson’s disease after differentiation into HLCs, when compared to the same mutation bearing Wilson’s patient-derived HLCs. Finally, the potential use of the Wilson’s mutation-introduced HLCs for drug screening was evaluated by treating current therapeutic agents of Wilson’s disease.

## 2. Materials and Methods

### 2.1. Cell Culture

hESCs (BG01 hESCs, WiCell, WI, USA) were stably maintained using feeder-free cell culture systems. In brief, hESCs were cultured on the top of Matrigel (Becton Dickinson, NJ, USA)-coated cell culture dishes and fed with fresh mTESR1 (Stemcell Technology, Vancouver, BC, Canada). hESCs were regularly passaged every three or four days using TryPLE (Thermo Fisher Scientific, Waltham, MA, USA) reagent. Wilson’s disease induced pluripotent stem cells (iPSCs) generated from patient fibroblast were kindly provided by Dr. Yong-Mahn Han from Korea Advanced Institute of Science and Technology (KAIST). Primary human hepatocytes (PHHs, Lot No.303-Caucasian, BD Biosciences, Franklin Lakes, NJ, USA) were cultured on the top of collagen-I coated dishes and maintained using Hepatocyte Culture Medium (Corning Inc., Corning, NY, USA).

### 2.2. Gene Editing

To introduce R778L mutation, guide RNA targeting specific locus of *ATP7B* was cloned into pSpCas9(BB)2APuro (PX459) V2.0 vector, which was a gift from Feng Zhang (Addgene, MA, USA, plasmid #62988). One μg of cloned guide RNA vectors and 20 pmole of single-stranded DNA oligonucleotides that contain mutated sequence were co-transfected into BG01 hESCs using Lipofectamine™ 3000 Transfection Reagent (Thermo Fisher Scientific). Two days after transfection, transfected BG01 were treated with 0.5 μg/mL of puromycin for 24 h to get rid of non-transfected cells. Single colonies obtained from transfected BG01 were individually sequenced, and the mutated colony was selected for further analysis. The Oligo information is listed in Table S1.

### 2.3. Embryonic Body Generation

To generate embryonic body, hESCs and hiPSCs were treated with dispase (Stemcell Technology) and subsequently transferred as clumps into petri dish. To induce spontaneous differentiation, embryonic body was maintained for 7 days with 10% Fetal Bovine Serum (FBS, Thermo Fisher Scientific) in Dulbecco’s Modified Eagle Medium (DMEM)/F12 medium (GE Healthcare Life Sciences, Chicago, IL, USA). Then, embryonic body was attached into Matrigel-coated dishes, which was differentiated for additional 7 days.

### 2.4. Differentiation into Hepatocyte-Like Cells (HLCs)

Differentiation of undifferentiated hPSCs into HLCs was performed as described previously [25]. In brief, hPSCs were differentiated into definitive endoderm by the combinatorial treatment of activin A (R&D Systems, Minneapolis, MN, USA) and CHIR99021 (Tocris Bioscience, Bristol, UK) and further induced into mature HLCs by treatment of bone morphogenetic protein 2 (BMP2), fibroblast growth factor 2 (FGF2), and hepatocyte growth factor (HGF) (all from R&D Systems), and Dex (Sigma-Aldrich, St. Louis, MO, USA).

### 2.5. Immunostaining

Differentiated HLCs were fixed in 4% Paraformaldehyde (PFA, Bio Solutions, Seoul, Korea) and then permeabilized using 0.1% Triton X-100 (Sigma-Aldrich) in Phosphate-Buffered Saline (PBS, GE Healthcare Life Sciences). After blocking with 4% Bovine Serum Albumin (BSA, Bovogen Biologicals, Melbourne, Australia) in PBS, cells were stained with human-specific antibodies against albumin (ALB, Sigma-Aldrich) and cytoskeletal 18 (CK18, Agilent Technologies, Santa Clara, CA, USA). Fluorescence-conjugated secondary antibodies (Thermo Fisher Scientific) and 4′,6-diamidino-2-phenylindole (DAPI, Sigma-Aldrich) were used for the nuclear visualization of the stained specimen.

### 2.6. Flow Cytometry

Before intracellular staining, differentiated HLCs were dissociated using TrypLE (Thermo Fisher Scientific) reagent. Cells and then fixed and permeabilized with Cytofix/Cytoperm™ (Becton Dickinson, Franklin Lakes, NJ, USA) following the manufacturer’s guidance and instructions. Antibodies against ALB and hepatocyte nuclear factor 4α (HNF4A, Abcam, Cambridge, UK) were used to quantify the differentiation efficiency. Labeled primary antibodies were indirectly visualized using fluorescence-conjugated secondary antibodies. Data were acquired for each sample using a NovoCyte flow cytometer (ACEA Biosciences, San Diego, CA, USA).

### 2.7. Real-Time PCR

Cells were lysed by TRIzol™ reagent (Thermo Fisher Scientific), and isolation of RNA was performed by following the manufacturer’s guidance and instructions. For cDNA synthesis, 1 μg of total RNA was annealed with 200 ng random hexamer and then incubated with RevertAid H Minus Reverse Transcriptase (Thermo Fisher Scientific) to synthesize cDNA. To compare the relative gene expression level, CFX-Connect real-time system (Bio-Rad Laboratories, Hercules, CA, USA) was used and the relative expression level of each gene was analyzed using a comparative threshold cycle method. *GAPDH* gene was used to normalize the expression levels of genes of interest, and the primer information used in this study is listed in the Table S2.

### 2.8. Periodic Acid-Schiff (PAS) Staining

To visualize glycogen storage, cells were washed with PBS and fixed using 4% PFA solution for 10 min at room temperature (RT). After additional washing with PBS, 0.5% periodic acid solution (Merck Millipore, Burlington, MA, USA) was used to generate aldehyde for 20 min at RT. Cells were rinsed with PBS and then reacted with Schiff’s reagent (Merck Millipore) for 30 min at RT. These serial reactions produce bright magenta coloration in areas of cells containing glycogen that is observed under the optical microscope.

### 2.9. Cell Viability Assay

To measure cell viability, Cell Counting Kit-8 (CCK-8, Dojindo Molecular Technologies, Rockville, MD, USA) was utilized. Briefly, HLCs were plated onto 96-well plates at a density of 1 × 10^5^ cells per well. After aspirating culture medium, cells were incubated with CCK-8 solution diluted in DMEM/F12 without phenol red for 30 min. After incubation, optical density values at 450 nm (OD 450) and 600 nm (OD 600) were measured by SpectraMax iD3 (Molecular Devices, San Jose, CA, USA) and OD 600 was subtracted from OD 450 to remove turbidity effect.

### 2.10. Chemical Treatment

To generate Wilson’s disease environment, copper chloride (Sigma-Aldrich) was supplemented to culture medium in various concentrations. D-penicillamine, trientine hydrochloride, bathocuproinedisulfonic acid, and DL-α-tocopheryl acetate (all from Sigma-Aldrich) were additionally treated to investigate recovery effect.

### 2.11. Measuring Copper Concentration

Intracellular copper concentration was detected by copperGREEN. (Goryo chemical, Sapporo, Japan). Hepatocytes were plated onto 96 black well plates at a density of 1 × 10^5^ cells per well. Before copperGREEN reaction, cells were rinsed with PBS containing 200 μM Ethylenediaminetetraacetic Acid (EDTA, Thermo Fisher Scientific) to remove extracellular copper ion. Then, cells were incubated with 5 μM of copperGREEN diluted in cell culture medium for 3 h. After several washing steps, fluorescence was measured by SpectraMax iD3 with an excitation wavelength of 470 nm and an emission wavelength of 510 nm.

### 2.12. RNA Sequencing

For RNA-seq analysis, we prepared mRNA sequencing libraries as paired-end reads with a length of 100 bases using the TruSeq RNA Library Preparation Kit (Illumina, San Diego, CA, USA) according to the manufacturer’s guidance and instructions. The libraries were sequenced as paired-end reads (2 × 100 bp) using the HiSeq 2500 (Illumina). Differential expression analysis was performed by Cuffdiff, a tool to estimate differential expression at gene and transcript levels. To enhance the analysis accuracy, multi-read-correction and frag-bias-correct options were applied. All other options were set to default values. Differentially expressed genes were identified based on the *q* value threshold less than 0.05 for correcting errors caused by multiple testing.

### 2.13. Statistical Analysis

Data are presented as means of three independent experiments. The statistical significance of the real-time PCR data and other assays was evaluated by Student’s t-test, and *p* < 0.05 was considered significant.

## 3. Results

### 3.1. Generation of Wilson’s Disease Model Using the CRISPR/Cas9 System

**Figure 1:**
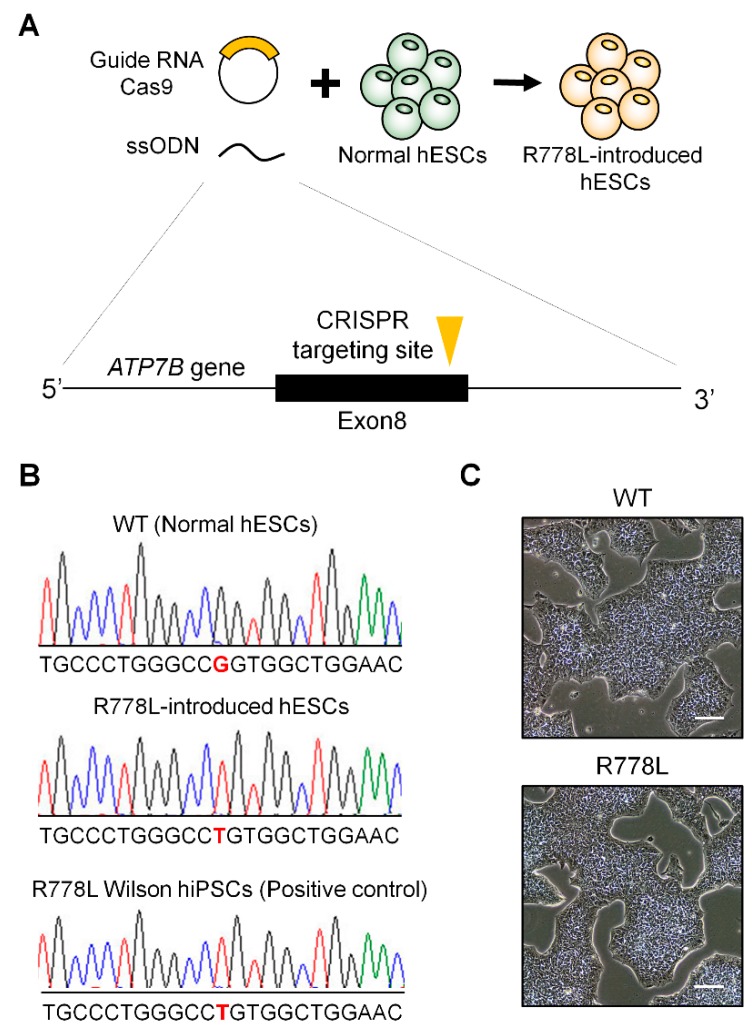
Introduction of R778L mutation into normal human embryonic stem cells (hESCs): (**A**) Graphical strategy showing the whole process of genetic model establishment harboring R778L mutation in *ATP7B* using Clustered Regularly Interspaced Short Palindromic Repeats (CRISPR)/Cas9 system. To introduce R778L mutation, hESCs were transfected with specifically designed single-guide RNAs (sgRNAs) and single-stranded oligodeoxynucleotides (ssODNs), simultaneously. (**B**) Sequencing analysis to verify successful genome editing: Single nucleotide substitution (c.2333G>T, Substitution, position 2333, from G to T) was confirmed in R778L-introduced hESCs. (**C**) Representative images of wildtype (WT) and R778L-introduced hESCs: R778L-introduced hESCs did not disturb the classical morphology of hESCs, a cobblestone shape with a clear boundary. Scale bar, 100 µm. WT, WT hESCs; R778L, R778L-introduced hESCs.

To model Wilson’s disease, we targeted exon 8 of *ATP7B* gene to introduce the R778L (c.2333G>T) mutation in wildtype (WT) hESCs. The overall strategy of CRISPR/Cas9 targeting is described in Figure 1A. Among various single-guide RNA (sgRNA) candidates which target the adjacent genomic region of R778L mutation locus, we selected two sgRNAs considering the probability of off-target and spatial proximity to the target region. Two sgRNAs were incorporated into px459 plasmid that co-expressed Cas9 protein and puromycin resistance gene. In order to introduce single-nucleotide substitution for R778L, we designed single-stranded oligodeoxynucleotides (ssODNs) that contain mutational sequence (c.2333G>T) and homologous sequences on each side of the target region.

To enhance the cloning efficiency, non-transfected hESCs were excluded by puromycin selection after the transfection of sgRNA-cloned px459 and ssODNs. Puromycin-selected hESCs were subsequently cultured in hESC culture condition and cloned as a single cell by serial dilution to get a clonal population. To confirm the genetic mutation of the clonal population, genomic DNA was extracted from each clone and sequenced the targeting region after polymerase chain reaction. Among various clonal populations, one clone from sgRNA-2-transfected groups was confirmed for the homozygous substitution of G to T at c.2333 locus (Figure 1B). In addition, possible off-target sites of the sgRNA used in this study were located at the noncoding regions, and there were no unwanted mutations on the top three predicted off-target sites (Figure S1 and Table S3). This R778L mutation-introduced clone (R778L-introduced) showed classical morphology of hESCs and was positive for hESC markers such as octamer-binding transcription factor 4 (OCT4), homeobox protein NANOG (NANOG), SRY-box 2 (SOX2), and stage-specific embryonic antigen 4 (SSEA4) confirming self-renewal ability (Figure 1C and Figure S2). Spontaneous differentiation also showed that R778L mutation did not affect differentiation capability (Figure S3). R788L mutation-bearing Wilson’s disease patient-derived hiPSCs (Wilson hiPSCs; Figures S2 and S3) was used as a control to confirm the correct introduction of R778L mutation in WT hESCs and the effectiveness of R778L-introudced hESC for modeling Wilson’s disease in the entire experiments in this study.

### 3.2. Differentiation of WT, R778L-Introduced hESCs, and Wilson iPSCs into Hepatocyte-Like Cells (HLCs)

**Figure 2:**
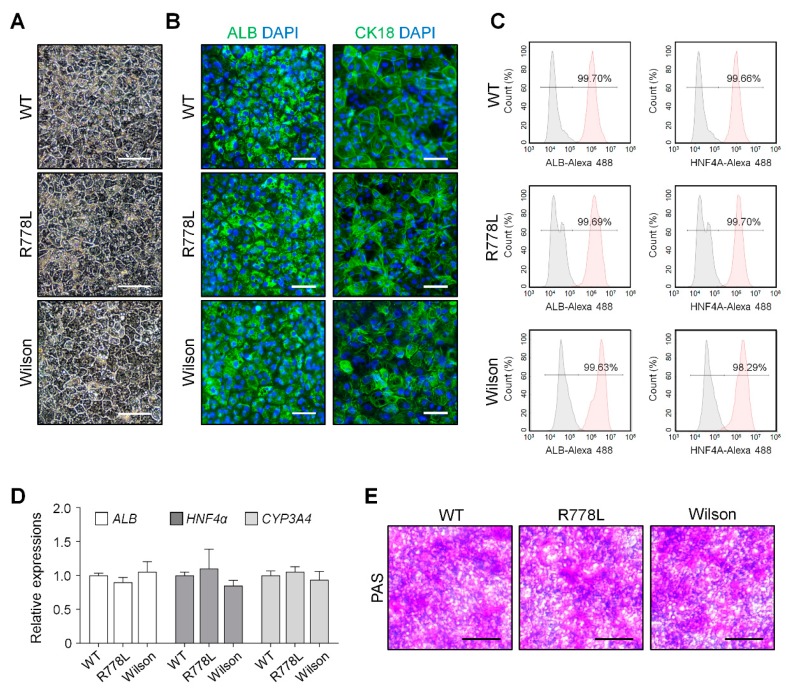
Hepatic differentiation of WT hESCs, R778L-introduced hESCs, and Wilson hiPSCs: (**A**) Representative images of WT-HLCs and R778L-introduced HLCs, and Wilson hiPSC-HLCs. Differentiated HLCs hepatocytes are arranged in hexagonal morphology. Scale bar, 100 µm. (**B**) Immunostaining of albumin (ALB, cytosolic protein) and cytoskeletal 18 (CK18, plasma membrane protein) was performed in differentiated HLCs. DAPI (4′,6-diamidino-2-phenylindole) showed nuclear counterstaining (blue). Scale bar, 50 µm. (**C**) Flow cytometric analysis on the expression levels of ALB and hepatocyte nuclear factor 4α (HNF4A). Differentiated HLCs exhibited high purity (>99% of ALB and HNF4A-positive cells). (**D**) Relative expression of hepatocyte-specific genes *ALB*, *HNF4A*, and *CYP3A4* in differentiated HLCs. Expression levels were determined by real-time PCR, normalized by *GAPDH*, and expressed as a fold change relative to the expression level in WT. The data are presented as the mean ± SE of three independent experiments. (**E**) Representative images of Periodic Acid-Schiff (PAS) staining in WT-HLCs, R778L-introduced HLCs, and Wilson hiPSC-HLCs. Glycogen storage assessed by PAS staining showed a similar level. Scale bar, 100 µm. WT, WT-derived HLCs; R778L, R778L-introduced-derived HLCs; Wilson, Wilson hiPSC-derived HLCs.

We differentiated WT, R778L-introduced, and Wilson hiPSC into hepatocyte-like cells (HLCs) to analyze the feasibility of in vitro modeling of hESC-derived Wilson’s disease. By following our previously published protocol [25], we carried out the whole differentiation process which is separated by several developmental stages such as definitive endoderm, hepatoblast, immature hepatocyte, and maturation. After finishing the differentiation process, WT-HLCs, R778L-introduced HLCs, and Wilson hiPSC-HLCs exhibited classical hexagonal-shaped morphology (Figure 2A) and immunostaining confirmed that all three differentiated HLCs (WT-HLCs, R778L-introduced HLCs, and Wilson hiPSC-HLCs) strongly expressed human ALB and CK18, the main cellular markers for hepatocytes (Figure 2B). The purity of the differentiated HLCs further assessed by flow cytometry and demonstrated that over 99% of the cells were positive for hepatic markers, ALB, and HNF4A in WT-HLCs, R778L-introduced HLCs, and Wilson hiPSC-HLCs (Figure 2C). Real-time PCR analysis of *ALB*, *HNF4a*, and *CYP3A4* (Figure 2D); RNA sequencing for hepatic marker; transcription factor; drug metabolism enzyme; and drug transporter (Figure S4) and PAS staining (Figure 2E and Figure S5) further demonstrated no differences in hepatic characteristics among the differentiated WT-HLCs, R778L-introduced HLCs, and Wilson hiPSC-HLCs. Together, these data indicate that introduction of R778L mutation did not affect the hepatic differentiation of the hESCs, confirming the potency of R778L-introduced HLCs to test the influence of the introduced mutation in hepatic model in vitro.

### 3.3. Comparing Vulnerability Against Copper in WT-HLCs, R778L-Introduced HLCs, and Wilson hiPSC-HLCs

**Figure 3:**
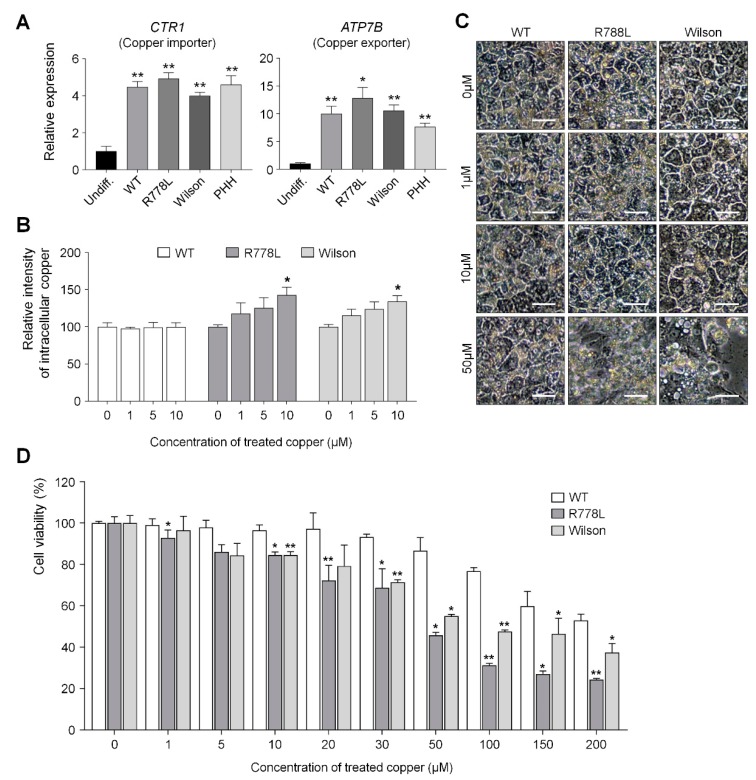
R778L-introduced HLCs show vulnerability against copper treatment as shown in Wilson hiPSC-HLCs. (**A**) Relative expression of copper-related genes *CTR1* and *ATP7B* in WT-HLCs, R778L-introduced HLCs, and Wilson hiPSC-HLCs: Expression levels were determined by real-time PCR, normalized by *GAPDH*, and expressed as a fold change relative to the expression level in Undiff. The data are presented as the mean ± SE of three independent experiments; * *p* < 0.05; ** *p* < 0.01. (**B**) The relative intensities of intracellular copper levels in differentiated HLCs: Fluorescence intensities measured using copperGREEN were represented in the graph as a relative value to the intensity level in 0 µM copper group of WT, R778L, and Wilson, respectively. The data are presented as the mean ± SE of three independent experiments; * *p* < 0.05. (**C**) Representative images of hepatocyte morphology against copper chloride supplementation: The toxicity caused by excessive copper disturbed morphology of hepatocytes. Scale bar, 50 µm. (**D**) Changes in the viability of HLCs against copper chloride supplementation. Fluorescence intensities measured using CCK-8 were represented in the graph as a relative value to the intensity level in 0 µM copper group of WT. The data are presented as the mean ± SE of three independent experiments; * *p* < 0.05; ** *p* < 0.01. Undiff, Undifferentiated WT hESCs; WT, WT-derived-HLCs; R778L, R778L-introduced-derived HLCs; Wilson, Wilson hiPSC-derived-HLCs; PHH, Primary human hepatocytes.

The relative expression level of *CTR1* (Copper importer) and *ATP7B* (Copper exporter) genes in WT-HLCs, R778L-introduced HLCs, and Wilson hiPSC-HLCs showed similar levels with primary human hepatocytes (PHH) (Figure 3A), which means that the introduction of R778L mutation did not affect the mRNA expression level of the copper transporter. However, although the accumulation of intracellular copper was not observed in the culture of WT-HLCs with copper chloride, the culture of R778L-introduced HLCs in the presence of copper chloride showed accumulation of intracellular copper in a dose-dependent manner as shown in the culture of Wilson hiPSC-HLCs (Figure 3B). This data indicates that the excretion of intracellular copper ion was defective in R778L-introduced HLCs like as in Wilson hiPSC-HLCs.

The main cause of hepatic failure in Wilson’s patients is hepatic toxicity caused by the disability of copper export from the liver [26,27]. In order to figure out this in the hESC-derived Wilson’s disease model, copper chloride was further supplemented in culture (from 0 μM to 50 μM) and the toxic effect of copper ion in the WT-HLCs and R778L-introduced HLCs were tested and compared with the copper toxicity in Wilson hiPSC-HLCs (Figure 3C). Treatment of copper chloride did not affect the morphological changes in WT-HLCs; however, massive cell death was detected in R778L-introduced HLCs at 50 µM of copper chloride treatment as shown in Wilson hiPSC-HLCs, indicating the high vulnerability against the copper ion was resulted from the introduction of R778L mutation in WT.

Cell viability analysis further supported this finding. When the copper chloride was treated in hepatocyte culture (ranging from 0 μM to 200 μM), the viability curve of WT-HLCs showed a consistent pattern up to the 20 μM-treated group and began to decline gradually in higher concentrations (Figure 3D). However, the decrease in cell viability was observed even at 1 μM concentration in the culture of R778L-introduced HLCs and the viability of R778L-introduced HLCs began to decline rapidly as like Wilson hiPSC-HLC culture when the copper concentration was increased in the culture (Figure 3D).

Whole transcriptome analysis further revealed that copper metabolism-related genes (copper ion transport, homeostasis, and binding) of R778L-introduced HLCs were differently expressed from WT-HLCs (Figure S6), although there are no differences in the hepatic characters as shown in Figure 2 and Figure S4. Moreover, gene ontology (GO) analysis of canonical pathway showed that pathways involved in induction and progression of liver fibrosis (Urokinase-type plasminogen activator and its receptor; uPA-uPAR pathway [28]), FGF pathway [29], Integrin pathway [30], EphirinB pathway [31], liver cell apoptosis (potassium ion channel pathway [32]), nonalcoholic fatty liver disease (Natural killer T; NKT pathway [33]), acute and chronic liver disease (inflammation pathway [34]), were enriched in R778L-introduced HLCs compared to WT-HLCs (Figure S6).

These results clearly showed that our R778L-introduced HLC model faithfully recapitulates disease phenotype of Wilson’s disease in vitro and encouraged us to further investigate the possibility of a cell-based platform for screening the efficacy of therapeutic agents.

### 3.4. Evaluation of In Vitro Wilson’s Disease Model for Drug Screening

We tested the model as a cell-based platform for assessing several therapeutic agents: D-penicillamine (DPA) is currently the most widely used copper chelating agents. DPA removes heavy metals like copper and iron by generating stable complexes, but sometimes, it had adverse effects [11,35,36]. Trientine hydrochloride (Trientine) emerged as an alternative for the patients who had DPA intolerance. Trientine has a similar mode of action but has a different clinical outcome when administrated to Wilson’s patients [27,36,37]. For another copper chelating agent, we chose bathocuproinedisulfonic acid (BCS) that is frequently used in many kinds of experiments to deplete copper ions. DL-α-tocopheryl acetate (vitamin E) known to relieve the oxidative stress was also selected because it might play a central role in the pathogenesis of Wilson’s disease by reducing oxidative stress [38,39].

**Figure 4:**
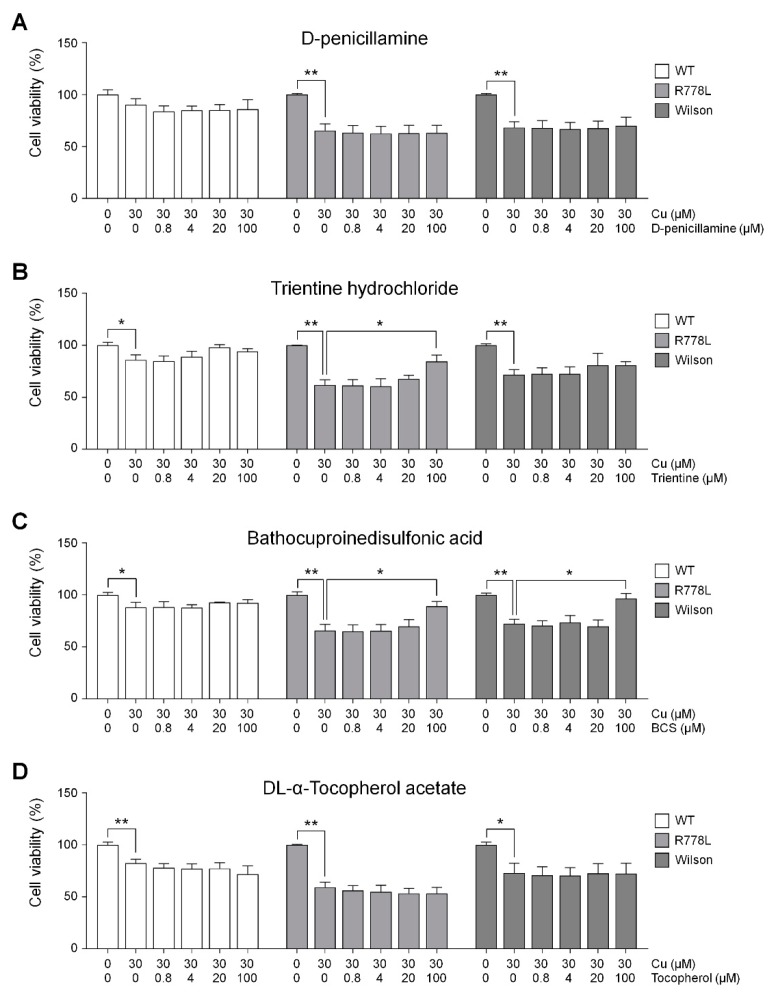
Efficacy tests of various therapeutic agents using Wilson’s disease model: Recovery outcomes of several copper chelating agents in the presence of copper chloride. Each graph represents the results of (**A**) D-penicillamine (DPA), (**B**) trientine, (**C**) bathocuproinedisulfonic acid (BCS), and (**D**) vitamin E. Fluorescence intensities measured using CCK-8 were represented in the graph as a relative value to the intensity level in untreated group of WT, R778L, and Wilson, respectively. The data are presented as the mean ± SE of three independent experiments; * *p* < 0.05; ** *p* < 0.01. WT, WT-derived-HLCs; R778L, R778L-introduced-derived HLCs; Wilson, Wilson hiPSC-derived-HLCs.

We conducted an experiment to find out the effect of those therapeutic agents in the culture of R778L-introduced HLCs with copper chloride, and the effects were compared to the those of Wilson hiPSC-HLCs. Selected therapeutic agents were treated at various concentrations from 0 μM to 100 μM with the 30 μM of copper chloride. Although DPA has been the most frequently utilized therapeutic agent, the cotreatment of copper chloride and DPA did not show any rescuing effect of copper-induced cell death in both R778L-introduced HLCs and Wilson hiPSC-HLCs (R778L; 65.9% ± 9.5%, Wilson; 69.9% ± 8.3%; Figure 4A). However, this result well corresponded to the previous study that DPA did not improve cell survival in a hepatoma cell line harboring R778L mutant [40]. Interestingly, in the case of trientine treatment, recovery effect exhibited a cell line-dependent manner. Even though the disease mutation of R778L-introduced HLCs and Wilson hiPSC-HLCs are identical, the rescuing cell survival was only observed in R778L-introduced HLCs (R778L; 88.4% ± 6.8%, Wilson; 80.7% ± 3.5%; Figure 4B). Recently, it was reported that the recovery effects of copper chelating agents were largely dependent on individuals. Comparative whole transcriptional analysis reveals differential gene expression between R778L-introduced HLCs and Wilson hiPSC-HLCs (Figure S7). Therefore, the different response against the trientine might have originated from the differences in genetic background between cell lines harboring the identical mutation. Otherwise, BCS consistently had the recovery effect in both R778L-introduced HLCs and Wilson hiPSC-HLCs (R778L; 92.5% ± 4.5%, Wilson; 96.7% ± 4.8%; Figure 4C), suggesting it efficiently chelated supplemented copper. However, antioxidant treatment did not seem to have much recovery effect in both groups when assessed by the results of vitamin E treatment group (R778L; 53.8% ± 8.5%, Wilson; 72.4% ± 10.0%; Figure 4D).

**Figure 5:**
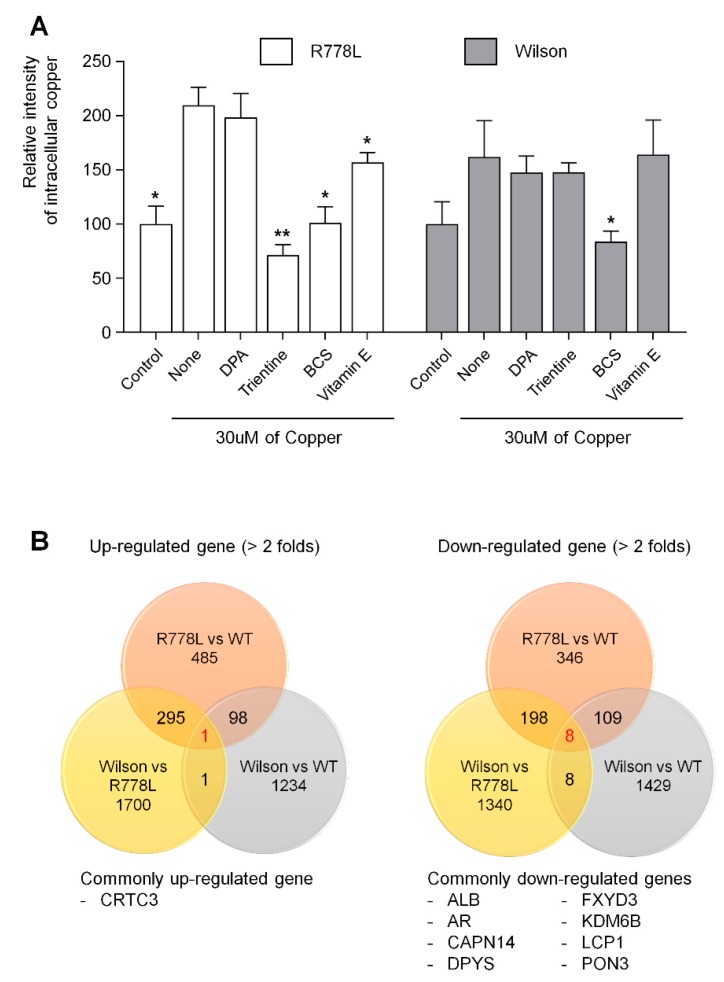
Analysis of intracellular copper levels and transcriptome of Wilson’s disease HLCs: (**A**) Several copper chelating agents were treated at a concentration of 100 μM in the presence of 30 μM of copper chloride. Fluorescence intensities measured using copperGREEN were represented in the graph as a relative value to the intensity level in control group of R778L and Wilson, respectively. The data are presented as the mean ± SE of three independent experiments; * *p* < 0.05; ** *p* < 0.01. WT, WT-derived-HLCs; R778L, R778L-introduced-derived HLCs; Wilson, Wilson hiPSC-derived-HLCs; DPA, D-penicillamine; Trientine, Trientine hydrochloride; BCS, bathocuproinedisulfonic acid disodium salt; Vitamin E, DL-α-tocopheryl acetate. (**B**) Analysis of commonly upregulated and downregulated genes in Wilson’s disease HLCs compared to WT-HLCs.

We reasoned that the recovery effect of copper chelating agents was a result of reduced intracellular copper concentration. To find the clue, we measured the intracellular copper concentration after recovery and confirmed the correlation between rescue of HLC viability and reduction of intracellular copper concentration in culture of R778L-introduced HLCs and Wilson hiPSC-HLCs with copper chloride. As shown that the BCS treatment rescued the copper-induced toxicity (Figure 4C) and reduced intracellular copper concentration (Figure 5A) in Wilson hiPSC-HLCs culture, the treatment of trientine and BCS increased the cell viability (Figure 4B,C) and also decreased the intracellular copper concentration (Figure 5A) in the culture of R778L-introduced HLCs with copper chloride. These data confirmed that the inhibition of copper-induced toxicity in R778L-introduced HLCs by copper chelating agents resulted from the reduction of intracellular copper concentration, indicating that the R778L-introduced HLCs were also applicable for drug screening against the copper chelators.

Lastly, we analyzed commonly upregulated and downregulated genes in Wilson’s disease-HLCs compared to WT-HLCs to find out future targeting for treating Wilson’s disease using the model established in this study (Figure 5B). In the comparison of whole transcriptome in R778L-introduced versus WT (484 genes were upregulated and 346 genes were downregulated), Wilson iPSCs versus WT (1234 genes were upregulated and 1429 genes were downregulated), and R778L-introduced versus Wilson iPSCs (1700 genes were upregulated and 1340 genes were downregulated), we found that only 1 gene (*CRTC3*: CREB-regulated transcription coactivator 3) was commonly up-regulated and 8 genes (*ALB*: albumin, *AR*: androgen receptor, *CAPN14*: calcium-activated neutral proteinase 14, *DPYS*: Dihydropyrimidinase, *FXYD3*: FXYD domain containing ion transport regulator 3, *KDM6B*: lysine demethylase 6B, *LCP1*: lymphocyte cytosolic protein 1, and *PON3*: paraoxonase 3) were downregulated in Wilson’s disease HLCs (Figure 5B). Manipulation of those commonly upregulated and downregulated genes might provide valuable information for further targeting Wilson’s disease.

**Figure 6:**
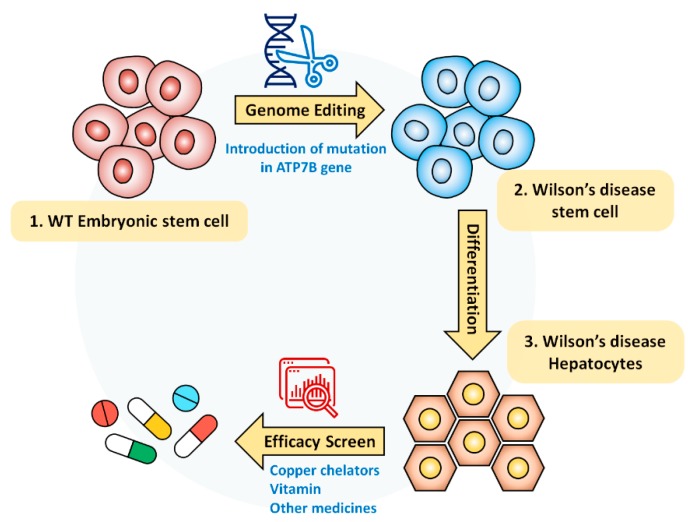
Schematic diagram of Wilson’s disease model for screening drug efficacy: hESC-derived Wilson’s disease model was generated by using CRISPR/Cas9 system. Established disease model could provide a rapid and reliable cellular model to assess the drug’s recovery efficacy and to screen personalized drugs before clinical application for Wilson’s disease patients.

Collectively, Wilson’s disease model in this study is not only effective to screen the efficacy of currently medicated drugs but also promising to study the future targeting of Wilson’s disease (Figure 6).

## 4. Discussion

Wilson’s disease patients mainly suffer from life-threatening liver disease failure, and several copper chelating agents have been considered as the first-line therapy to reduce the copper level. Although the effectiveness of copper chelating agents has been reported, the selection of chelators for treating each patient remains as an individual decision because patient-to-patient comparisons are not available [41,42].

In this study, we established an in vitro Wilson’s disease model from hESCs by introducing a mutation. The crucial point of in vitro disease modeling of Wilson’s disease is to recapitulate severe hepatic damages induced by excessive copper. The established R778L-introduced hepatocyte-like cells (HLCs) in this study exhibited higher vulnerability against excessive copper supplementation than WT-HLCs. This phenomenon also observed in Wilson hiPSC-HLCs, confirming that hESC-derived HLCs in this study precisely reproduced the detrimental condition of Wilson’s disease patient. Furthermore, our results in the following drug efficacy screening suggested that the curing effect of therapeutic agents was somewhat different depending on the genetic background. To offer optimal therapy, personalized drugs for an individual patient have been a major issue and patient-derived iPSCs is required for the best therapeutic efficacy. However, in case of extremely rare genetic disorders or any other disease where getting the cell source is hardly available, CRISPR-induced cell models would be a great alternative cellular model to study pathophysiology or general mechanisms since they share the general phenotype of the disease. Therefore, our strategy in this study would be one of the options to assess the clinical outcomes.

In addition to that, this study further suggested future targets for treating Wilson’s disease. From the whole transcriptome analysis, we found 1 upregulated gene and 8 downregulated genes in Wilson’s disease-HLCs, and these findings might provide invaluable information in future research for developing new therapeutics of Wilson’s disease. For example, several studies showed that the *CRTC3*, a commonly upregulated gene in Wilson’s disease HLCs of this study, is linked with obesity [43] and hepatic steatosis [44]. Because the fat accumulation is frequently observed in the liver of Wilson’s disease patients, targeting *CRTC3* possibly provides a new concurrent treatment with existing medications (e.g., copper chelators) for Wilson’s disease patients.

The established system in this study could be also used for predicting potential adverse effects of copper chelating agents. Treatment of DPA increases urinary copper excretion; however, many reports and articles claimed adverse effects of DPA, including bone marrow toxicity, elastosis cutis, nephrotoxicity, or lupus-like syndrome [45,46]. When a patient cannot take DPA any longer due to its adverse effects, trientine is used to cure Wilson’s disease. Although trientine has been shown to have fewer adverse effects than DPA, neurological deterioration had been reported in fewer patients who had trientine treatment [47,48]. Theologically, hESCs can be differentiated into any kind of cell type in our body. Thus, if the specific cells (e.g., neural cells, bone marrow cells, and renal cells) are generated from the established Wilson’s disease hESCs, we could screen effective dosages of therapeutic agents using this system [49,50,51].

Wilson’s disease model has been described elsewhere, but they mainly focused on basic characteristics such as differentiation capability or molecular behavior of ATP7B. To the best of our knowledge, this is the first report on generating Wilson’s disease model using the CRISPR/Cas9 system and on applying it to the drug efficacy screening. Therefore, the established model in this study could be useful not only for understanding pathophysiology of Wilson’s disease but also for screening drugs to treat Wilson’s disease as an alternative tool.

## 5. Conclusions

In this study, we generated a cellular model for Wilson’s disease (R778L mutation) from normal hESCs using CRISPR/Cas9 system. R778L mutation-introduced model was successfully differentiated into hepatocyte-like cells (HLCs), which indicates that Wilson’s disease model was successfully established from hESCs without the patient’s sample. Interestingly, R778L mutation-introduced HLCs exhibited higher vulnerability against excessive copper supplementation, resulting in more sensitive cytotoxicity against copper than wildtype HLCs. In the following screening experiment, several copper chelating reagents were treated to figure out the recovery efficacy and the effectiveness of the R778L mutation-introduced HLCs in drug screening was demonstrated. Taken together, we expect that the established Wilson’s disease model without patient’s sample might provide a new approach for screening drug’s recovery efficacy before the clinical application of certain drugs. Furthermore, this strategy might be applied to model various genetic disorders that have difficulties in obtaining patient samples.

## Supplementary Materials

The following are available online at https://www.mdpi.com/2073-4409/9/4/872/s1, Figure S1: OFF-target analysis in R778L-introduced hESCs; Figure S2: Expression of pluripotent genes in WT, R778L-introduced, and Wilson hiPSCs; Figure S3: Differentiation ability of WT, R778L-introduced, and Wilson hiPSCs; Figure S4: Comparative analysis of WT-HLCs, R778L-introduced HLCs, and Wilson hiPSC-HLCs; Figure S5: Quantification of PAS staining results of WT-HLCs, R778L-introduced HLCs, and Wilson hiPSC-HLCs; Figure S6: Gene expression profile analysis by gene set enrichment analysis assay between WT-HLCs and R778L-introduced HLCs, and Wilson hiPSC-HLCs; Figure S7: Genes differentially expressed between R778L-introduced HLCs and Wilson hiPSC-HLCs; Table S1: Oligo information used in CRISPR/Cas9 design; Table S2: Primer information used in real-time PCR; Table S3: Potential top twenty off-target sites are located at the non-coding region.
