# Supplementary material for: Human Embryonic Stem Cell-Derived Wilson’s Disease Model for Screening Drug Efficacy
Source: Cells. 2020 Apr 2;9(4):872. doi: 10.3390/cells9040872 (PMC7226780; doi:10.3390/cells9040872)
Supplement: Supplementary file 1 [file cells-09-00872-s001.pdf]

Human pluripotent stem cell-derived Wilson's disease model for screening drug efficacy

Table S1. Oligo information used in CRISPR/Cas9 design

Name	Oligo sequence
sgRNA-1	AGTGTTCCAGCCACCGGCCC
sgRNA-2	CATTGCCCTGGGCCGGTGGC
ssODNs	GGAGCCCTGTGACATTCTTCGACACGCCCCCCATGCTCTTTGTGTTCATTG CCCTGGGCCTGTGGCTGGAACACTTGGCAAAGGTAACAGCAGCTTCAGGT TCAGAAAAGAGCTGCTCCTTCAGTAAACAAATCTCACTTCCTCTGAACAC

Table S2. Primer information used in real-time PCR.

Target	Forward sequence	Reverse sequence
ALB	CACGCCTTTGGCACAATGAA	ATCTCGACGAAACACACCCC
HNF4A	ACTACATCAACGACCGCCAGT	ATCTGCTCGATCATCTGCCAG
CYP3A4	GGTGGTGAATGAAACGCTCAG	CACCCCTTTCCCAATGAACA
CTR1	TCCAACAGTACCATGCAACC	ATTGATCACCAAACCGGAAA
ATP7B	TTCCAGTGGATGGGAAAGTC	TCTGAGCCAAAGTGGTGTCA
GAPDH	CTCCTCCTGTTCGACAGTCA	TCACCTTCCCCATGGTGTCT

Table S3. Potential top twenty off-target sites are located at the non-coding region.

DNA	Chromosome	Position	Direction	Mismatches	Coding Region
CgTTGCCCTGGGCCtGTGGCCGG	chr16	2599589	+	2	No
CgTTGCCCTGGGCCtGTGGCCGG	chr16	2678042	+	2	No
CtTTGCCCTGGGCaGGTGGCAGG	chr17	28471799	+	2	No
aATTGCtCTGGGCaGGTGGCAGG	chr7	132190798	+	3	No
CAcTGgCCTGGGCaGGTGGCGGG	chr1	202010323	-	3	No
CATgGCaCTGGGCaGGTGGCAGG	chr2	56462179	+	3	No
CAaTGCCCTGGGtCaGTGGCTGG	chr2	121963488	+	3	No
CATTGCaCTGGGCaGGTGGgGGG	chr19	54484187	+	3	No
CATTGCCCgcGGCCGGgGGCCGG	chr21	13981602	-	3	No
CATTGCCCTGGGtgGGTGGgTGG	chr15	82304227	+	3	No
CAgaGCaCTGGGCCGGTGGCTGG	chr17	7438343	-	3	No
CATgGCCCTGGGgtGGTGGCGGG	chr17	9152501	-	3	No
aATTGCatTGGGCCGGTGGCAGG	chr17	39187138	-	3	No
CcTaGCCCTGGGCCGGgGGCAGG	chr10	102432490	+	3	No
CAcTGCCCTtGGCCGGaGGCAGG	chr9	137455329	+	3	No
CATTGtCCTGGGaaGcTGGCAGG	chr8	1479395	+	4	No
CATTGgCCaGGcaCGGTGGCAGG	chr8	20061954	-	4	No
CATTGgCCgGGGCCGGgGcCGGG	chr8	22599689	+	4	No
CATgGCtCTGGcCtGGTGGCTGG	chr8	48467444	+	4	No
CtTTGCCCTGGaCCGaTGGaGGG	chr8	80315223	+	4	No

Figure S1. OFF-target analysis in R778L-introduced hESCs.

<table>
<tr><th>Target</th><th>Chromosome</th><th>Position</th><th>Direction</th><th>Mismatches</th></tr>
<tr><td>crRNA: CATTGCCCTGGGCCGGTGGCNGG
DNA: aATTGCtCTGGGCaGGTGGCAGG</td><td>chr7</td><td>132190798</td><td>+</td><td>3</td></tr>
<tr><td colspan="5">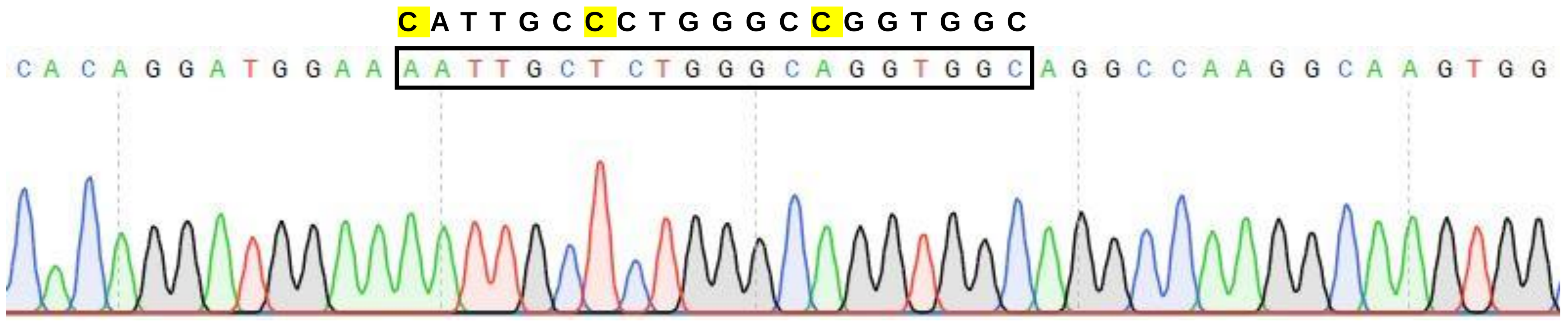
</td></tr>
<tr><td>crRNA: CATTGCCCTGGGCCGGTGGCNGG
DNA: CgTTGCCCTGGGCCtGTGGCCGG</td><td>chr16</td><td>2599589</td><td>+</td><td>2</td></tr>
<tr><td colspan="5">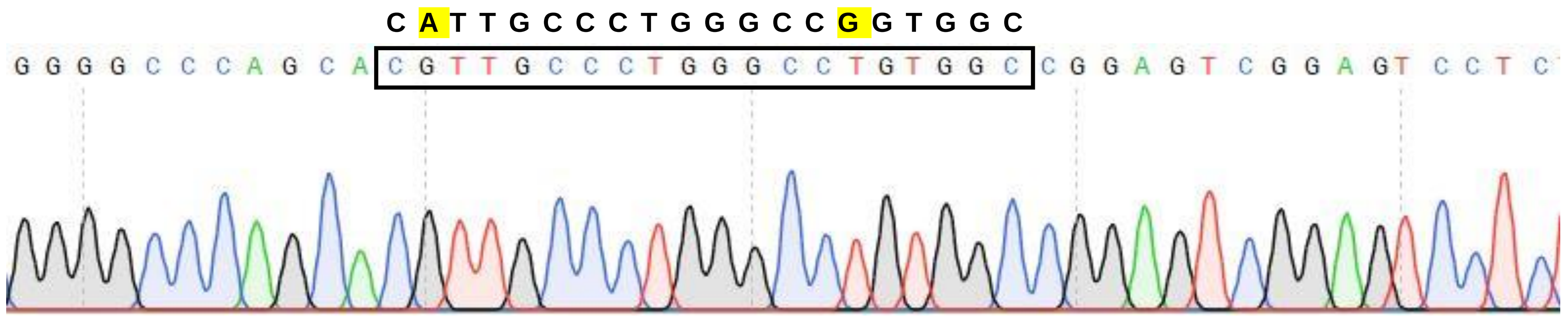
</td></tr>
<tr><td>crRNA: CATTGCCCTGGGCCGGTGGCNGG
DNA: CgTTGCCCTGGGCCtGTGGCCGG</td><td>chr16</td><td>2678042</td><td>+</td><td>2</td></tr>
<tr><td colspan="5">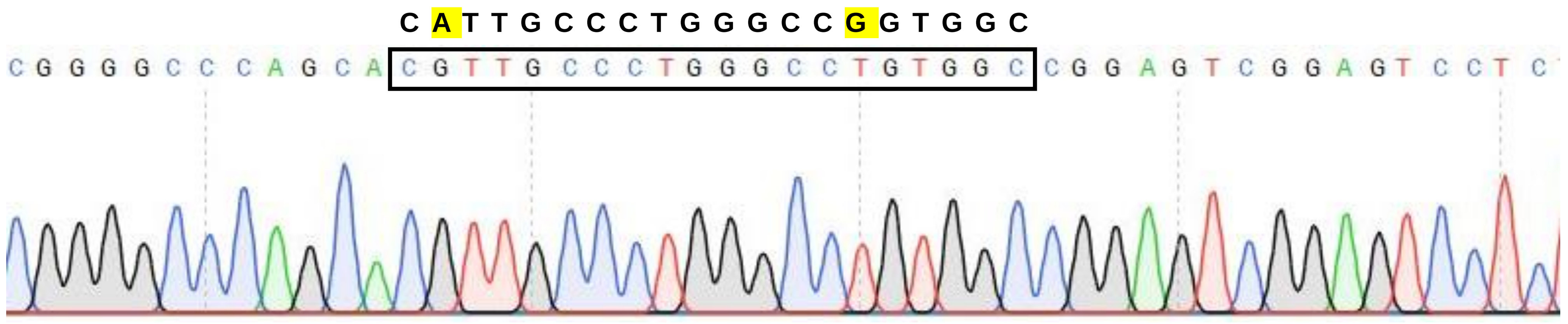
</td></tr>
</table>

The sequence analysis of the possible top three off-target sites for sgRNA-2 showed no off-target mutations.

Figure S2. Expression of pluripotent genes in WT, R778L-introduced and Wilson hiPSCs.

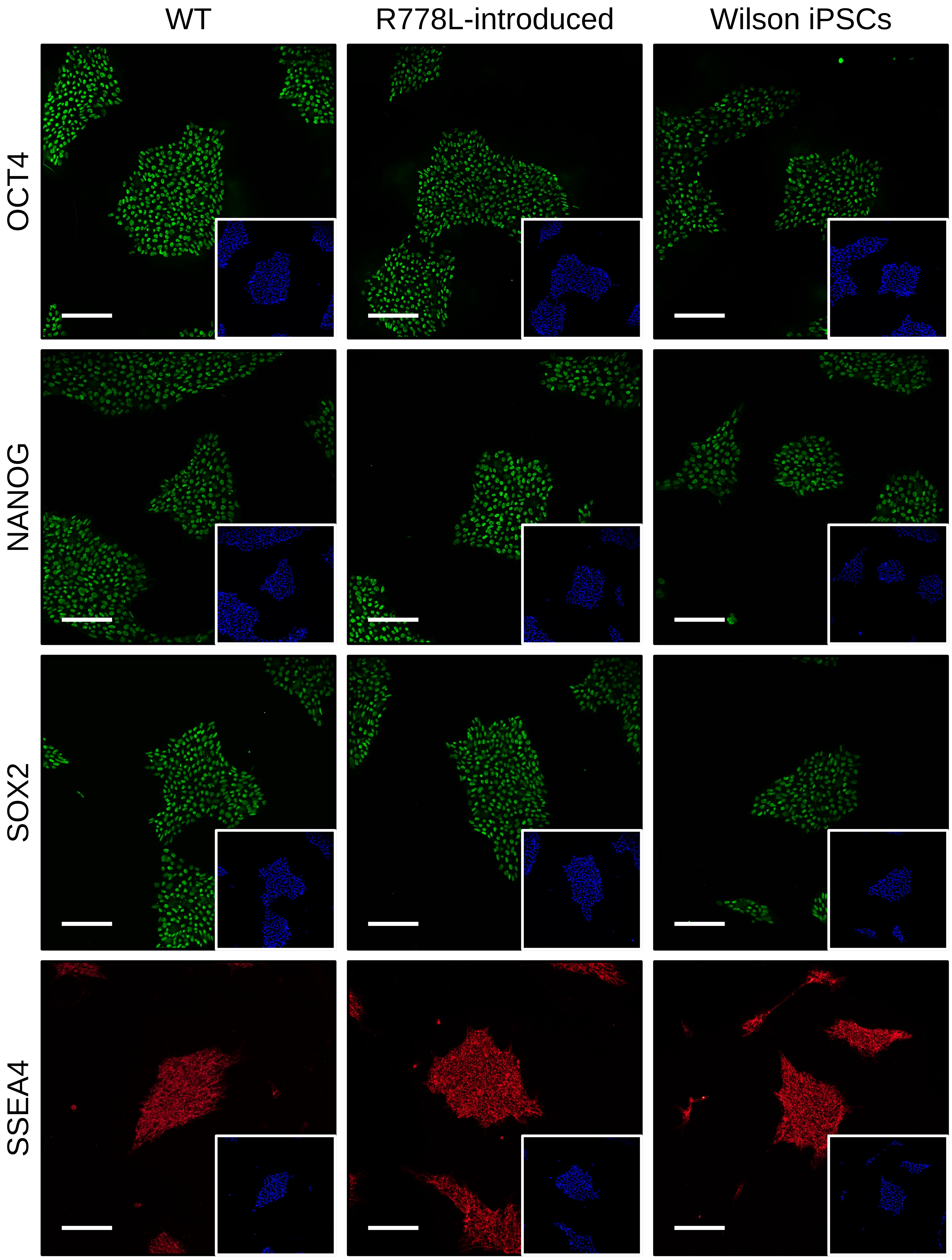

Immunostaining of OCT4, NANOG, SOX2, and SSEA4 was performed in WT hESCs, R778L-introduced hESCs and Wilson iPSCs. DAPI showed nuclear counterstaining (blue). Scale bar, 50 μm.

Figure S3. Differentiation ability of WT, R778L-introduced and Wilson hiPSCs.

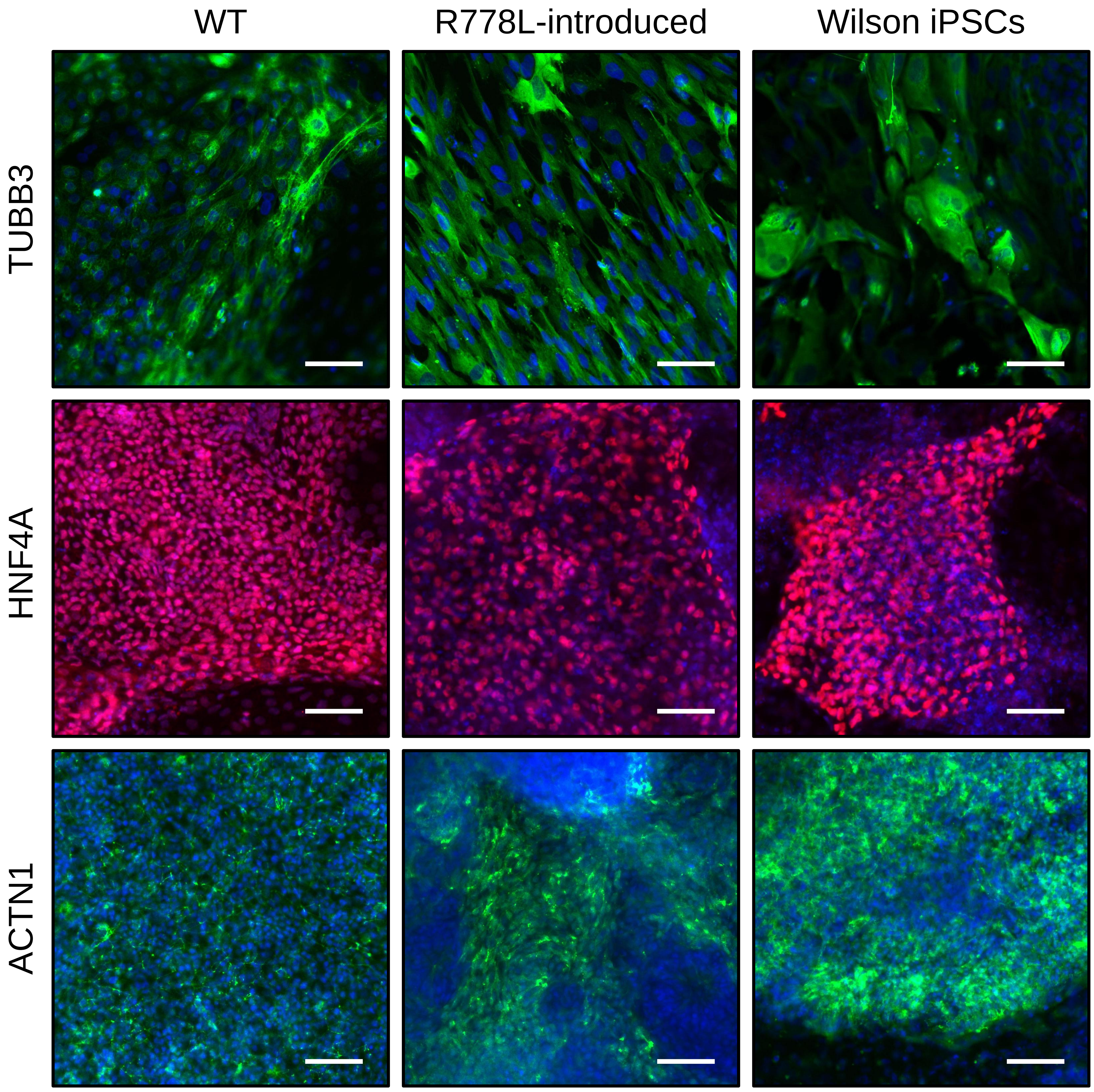

Immunostaining of TUBB3(Ectoderm marker), HNF4A(Endoderm marker), and ACTN1(Mesoderm marker) was performed in spontaneously differentiated cells from WT hESCs, R778L-introduced hESCs and Wilson iPSCs. DAPI showed nuclear counterstaining (blue). Scale bar, 100 μm.

Figure S4. Comparative analysis of WT-HLCs, R778L-introduced-HLCs and Wilson hiPSC-HLCs.

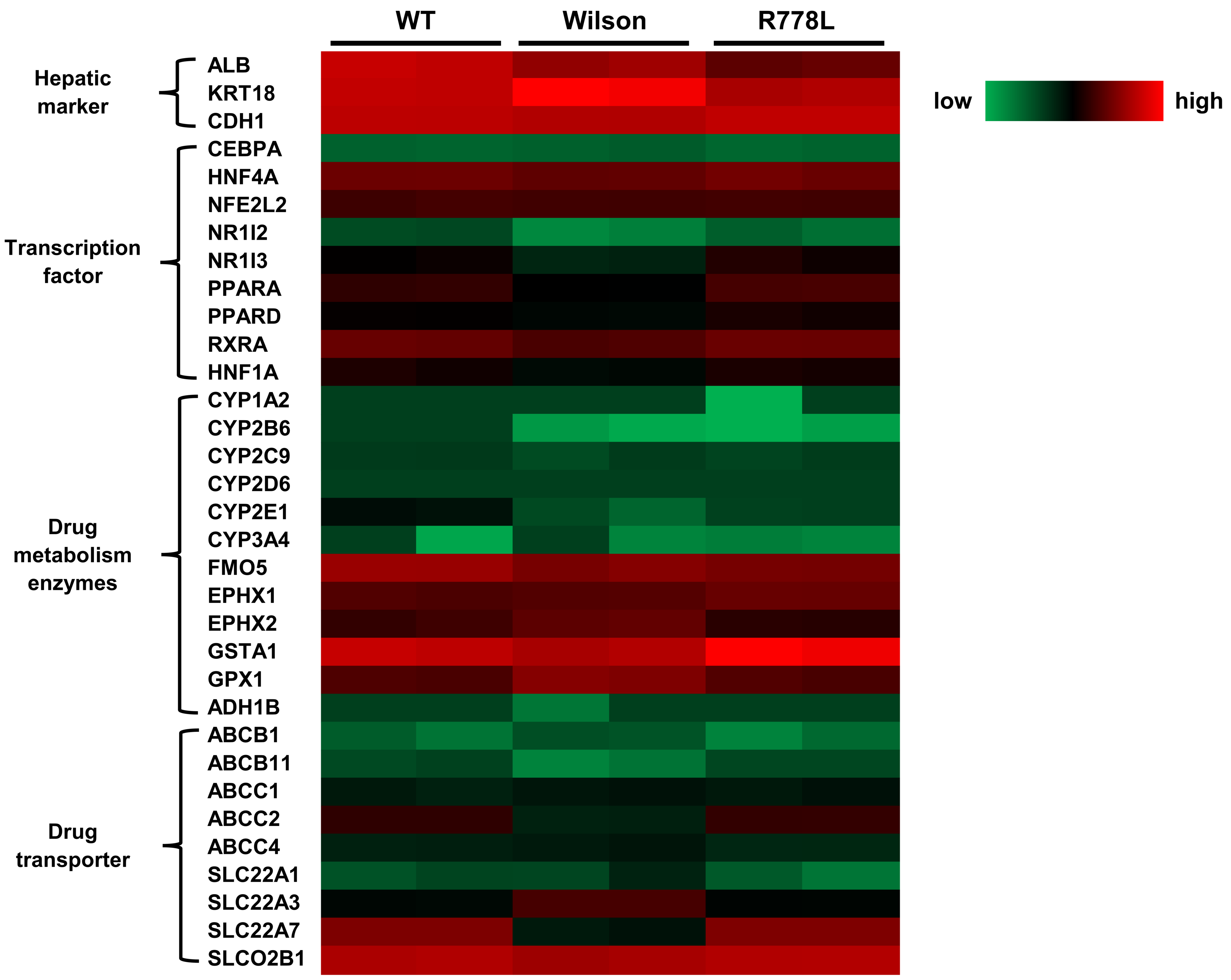

Comparative analysis of RNA seq for hepatic marker, transcription factor, drug metabolism enzymes, and drug transporter was represented as a heat map. These results demonstrated that there is no differences in hepatic characteristics among the differentiated WT-HLCs, R778L-introduced-HLCs, and Wilson hiPSC-HLCs

Figure S5. Quantification of PAS staining results of WT-HLCs, R778L-introduced-HLCs and Wilson hiPSC-HLCs.

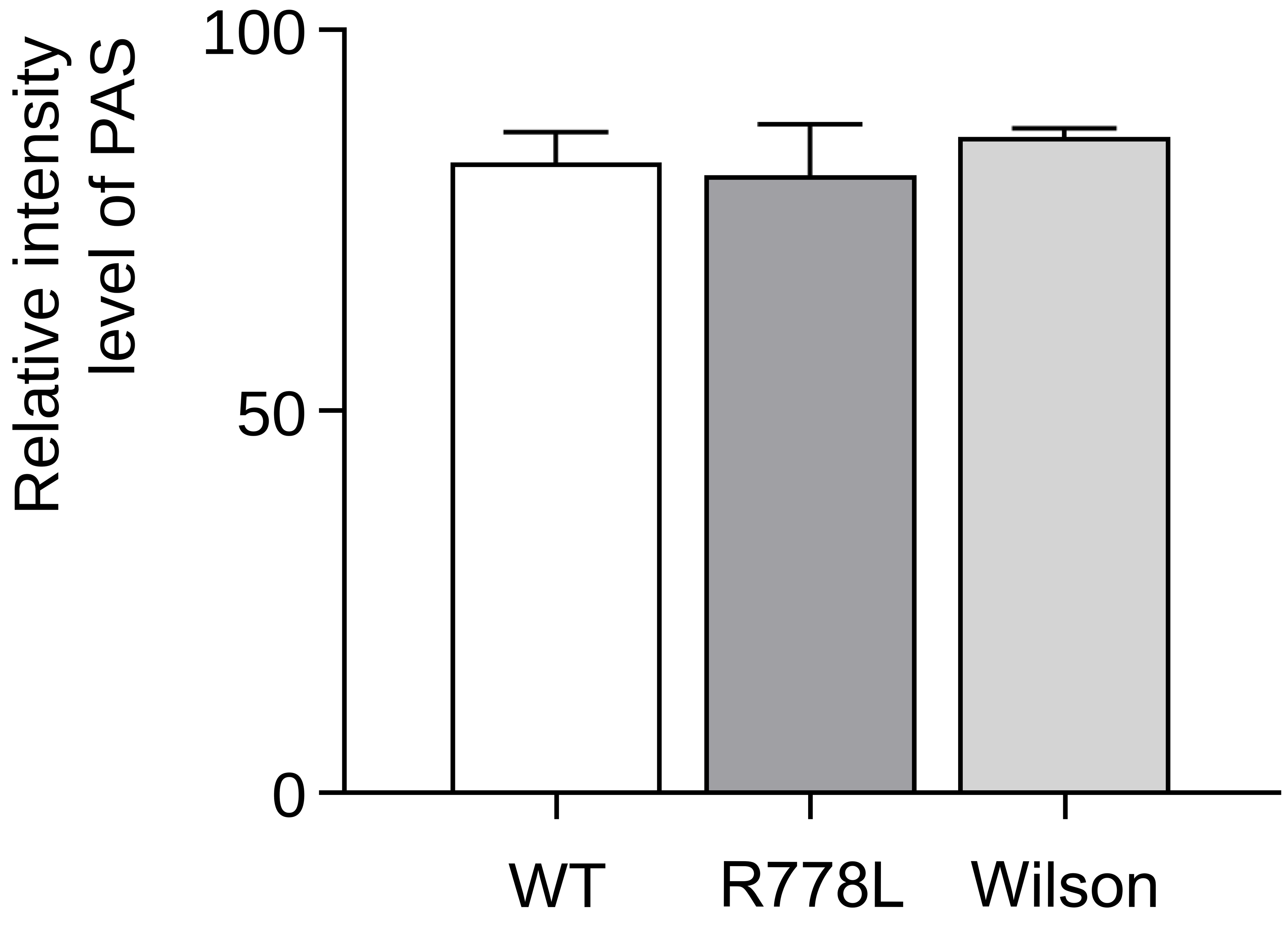

Stained region in PAS staining was quantified by image J tool and presented as the mean ± SE of three independent experiments; * $p < 0.05$; ** $p < 0.01$.

Figure S6. Gene expression profile analysis by gene set enrichment analysis assay between WT-HLCs and R778L-introduced-HLCs and Wilson hiPSC-HLCs.

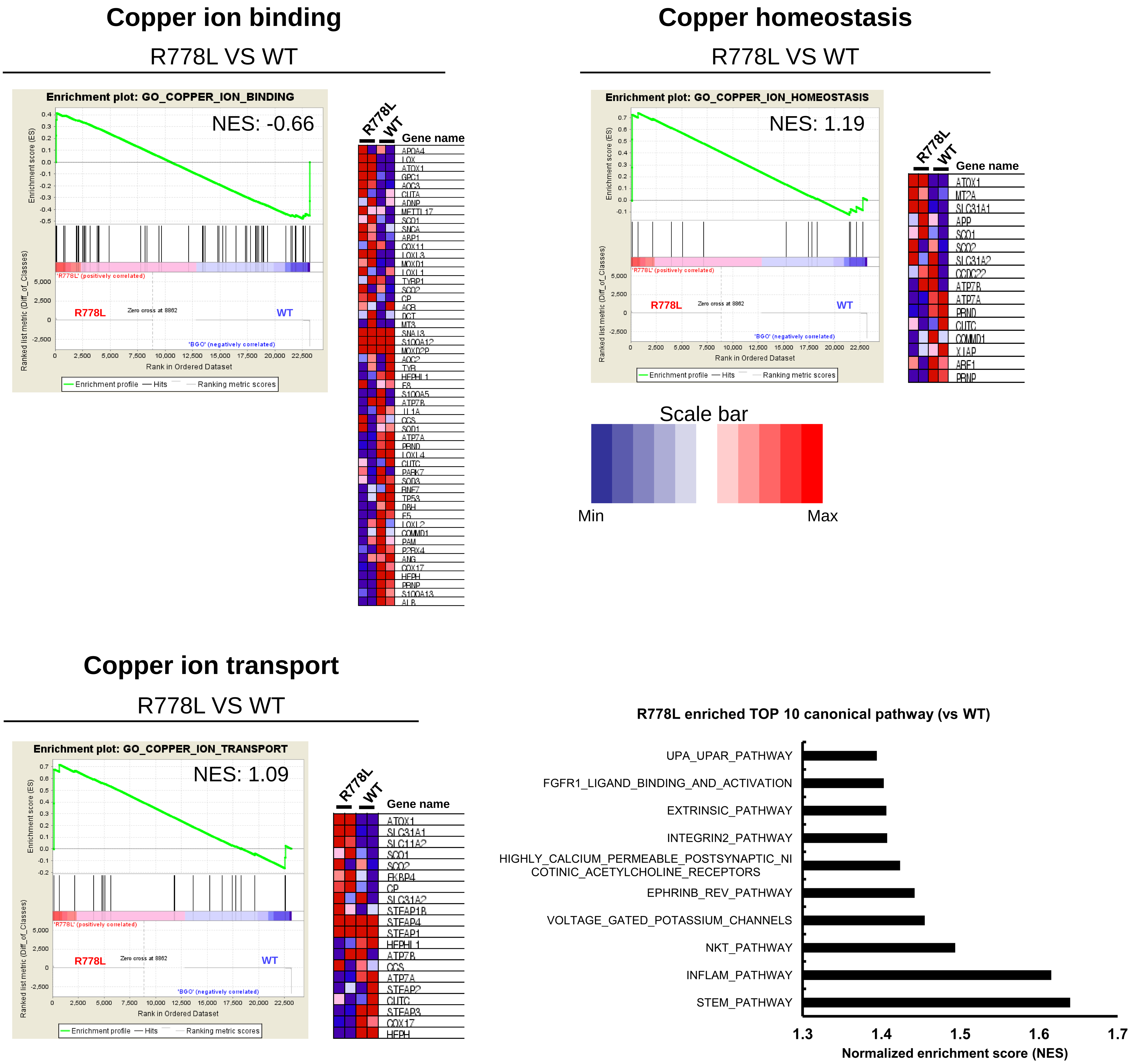

Heat map representation of the copper related genes in WT-HLCs and R778L-introduced-HLCs and Wilson hiPSC-HLCs. The heat-map was manipulated by "RANK METRIC SCORE" with Gene Set Enrichment Analysis software (GSEA v. 4.0.3). Furthermore, the enriched top 10 canonical pathways in R778L-introduced hepatocytes compared to WT hepatocytes was analyzed. NES: normalized enrichment score, Red: up-regulated; Blue: down-regulated.

Figure S7. Genes differentially expressed between R778L-introduced-HLCs and Wilson hiPSC-HLCs.

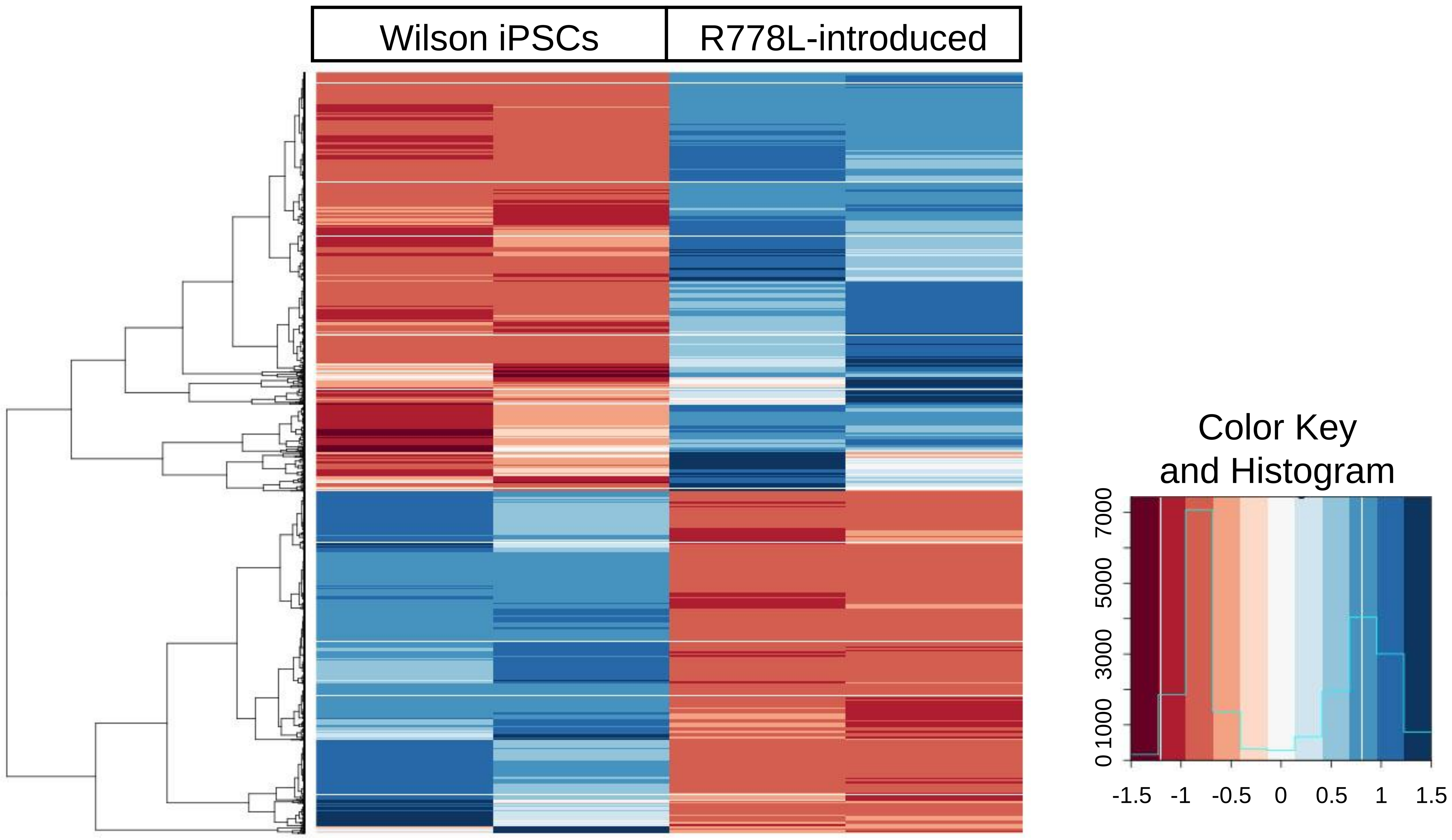

Differentially expressed genes in R778L-introduced-HLCs and Wilson hiPSC hepatocytes were represented as a heat map.
